# Neurobiology of functional (psychogenic) movement disorders

**DOI:** 10.1097/WCO.0b013e3283633953

**Published:** 2013-07-03

**Authors:** Mark J. Edwards, Aikaterini Fotopoulou, Isabel Pareés

**Affiliations:** a Sobell Department of Motor Neuroscience and Movement Disorders, UCL Institute of Neurology, Queen Square, London; b CEHP Research Department, UCL, 1–19 Torrington Place, London, UK

**Keywords:** conversion disorder, functional neurological symptoms, neurobiology, psychogenic

## Abstract

**Purpose of review:**

This review explores recent developments in understanding the neurobiological mechanism of functional (psychogenic) movement disorders (FMDs). This is particularly relevant given the resurgence of academic and clinical interest in patients with functional neurological symptoms and the clear shift in diagnostic and treatment approaches away from a pure psychological model of functional symptoms.

**Recent findings:**

Recent research findings implicate three key processes in the neurobiology of FMD (and by extension other functional neurological symptoms): abnormal attentional focus, abnormal beliefs and expectations, and abnormalities in sense of agency. These three processes have been combined in recent neurobiological models of FMD in which abnormal predictions related to movement are triggered by self-focused attention, and the resulting movement is generated without the normal sense of agency that accompanies voluntary movement.

**Summary:**

New understanding of the neurobiology of FMD forms an important part of reappraising the way that patients with FMD (and other functional disorders) are characterized and treated. It also provides a testable framework for further exploring the pathophysiology of these common causes of ill health.

## INTRODUCTION

It is common to begin review papers with a definition of the disorder under discussion. This presents some difficulties with the topic of this article: functional (psychogenic) movement disorders (FMDs). A common definition is that these are movement disorders presumed to be due to psychological factors or psychiatric illness and not structural or neurochemical disease [1]. There are difficulties with this definition however. First, the dichotomy between structural/neurochemical disease and psychological factors/psychiatric illness suggests a compartmentalized brain and mind: a concept not supported by centuries of scientific research. Second, the presumed psychological or psychiatric factors in FMD are not apparent in many patients [2,3], are present in many people without FMD, are not used by neurologists in the diagnosis [4], and have even been rejected by psychiatrists as a necessary diagnostic criterion in the equivalent psychiatric diagnosis, conversion disorder [5]. As we have argued previously [6], we prefer to define this disorder on the basis of its clinical appearance as a movement disorder that is significantly altered by distraction or nonphysiological manoeuvres (including dramatic placebo response) and which is clinically incongruent with movement disorders known to be caused by neurological disease.

## FROM CLINICAL CHARACTERIZATION TO MECHANISM

One of the key developments in recent years in FMD has been the refinement of diagnostic criteria to focus more on positive physical signs and investigation findings to support the diagnosis, rather than the presence of psychological distress [7–9]. This is one of the most important reasons why patients with FMD (and those with functional weakness) are such an important model for studying the neurobiology of functional neurological symptoms in general. In contrast to patients with symptoms such as sensory loss, pain, fatigue and memory disturbance, patients with FMD have objective motor signs that are amenable to clinical and experimental measurement. Diagnosis can therefore be positive in many patients, relying on important clinical differences in the way the movement disorder changes with physical examination manoeuvres, and provides a degree of certainty about the diagnosis, which may not be achievable in those whose symptoms are only measurable via self-report. From an experimental perspective, FMD can be objectively measured (e.g., using an accelerometer to measure tremor, using electromyography to measure reaction time or pattern of muscle recruitment), providing an objective parameter for experimental studies. Perhaps most importantly, the manner in which FMDs differ from typical ‘organic’ movement disorders both constrains and informs neurobiological models for symptom generation. Given the common co-occurrence of different functional neurological symptoms in the same patients, study of the neurobiology of FMD is likely to be directly relevant to the neurobiology of all functional neurological symptoms.

**Box 1:**
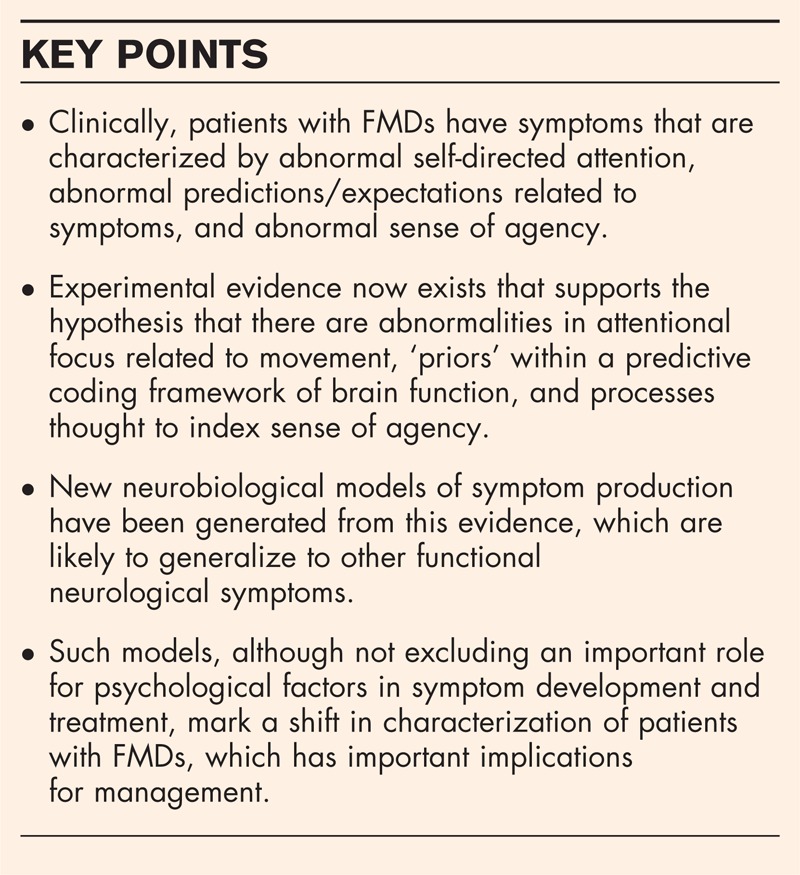
no caption available

## KEY CONCEPTS IN THE NEUROBIOLOGY OF FUNCTIONAL MOVEMENT DISORDERS

Following from modern diagnostic criteria for FMD, three key concepts emerge that must be encompassed by any neurobiological model that seeks to explain the disorder. The first relates to attention. A key feature that distinguishes patients with FMD from those with ‘organic’ movement disorders is that the FMD requires attention to manifest: when attention is distracted, there is typically a reduction, even disappearance of the movement disorder [7]. Conversely, during examination, movements are often performed with considerable visual attention towards movement [10]. The second key concept is symptom-related beliefs/expectations. This does not just mean consciously reportable beliefs about symptoms, but includes expectations or ‘priors’ in the setting of active inference in the brain. Active inference refers to a well grounded neurobiological theory of brain function that the brain actively predicts and seeks to explain sensory input on the basis of past experience: there is an internal model of the world that is used to interact with and explain the world [11]. The interaction between ‘bottom-up’ sensory information and ‘top-down’ predictions about that information is suggested to take place at multiple levels of an interconnected hierarchy, and the end result of this process is a percept or movement. Patients with FMD can present symptoms that are incongruous with basic neuroanatomical/neurophysiological constraints of disease, but which, conceivably, fit with reasonable lay beliefs about brain function. For example, when the tremoring limb of patients with functional tremor is restrained, tremor often spreads to other body parts. This is something that defies some basic principles of organic tremor generation. An example of the same phenomenon outside FMD is of tubular visual field defect in which patients report a visual field defect that does not change in size when the patient is examined close to them or far away – something that defies the laws of optics. In this way, FMD can be characterized as being shaped by ‘high-level’ beliefs about symptoms. The third key concept relates to agency. Agency refers to a fundamental aspect of human self-consciousness. We can make reportable judgements about whether we did something (’I moved my arm’ vs. ‘My arm was moved’) and in a more real-world context, we possess a background subjective sense of control over our actions – a sense of agency [12]. The abnormal movements in FMD look like movement that has been deliberately (i.e. with conscious fore-thought) produced by the patient because attention is required for the movement to manifest and the movement produced is not congruent with some basic neuroanatomical/physiological constraints. Such movements would be predicted to be associated with a strong sense of agency. However, the self-report of patients is that the movement is not under their control, and the implication of this is that there is likely to be a disruption of processes in patients with FMD that would usually imbue such movements with a sense of agency.

Below we review recent work in relation to patients with FMD and these three key concepts before discussing the recent attempts to construct a neurobiological framework to explain FMD, and by extension, other functional neurological symptoms.

## ATTENTION

Recent work has, in an experimental context, probed the clinically well described phenomenon in FMD of improvement in symptoms with distraction, and the converse worsening of symptoms and even the production of completely new symptoms when attention is drawn towards the body, typically during physical examination. Pareés *et al.*[13^▪^] looked at reaction and movement times in a group of patients with FMD in different paradigms designed to manipulate the degree of predictability of the movement (and hence its capacity for preplanning). Performance on a ‘one-back’ task, in which subjects had to move a cursor to a target seen in a previous trial, was abnormal in FMD patients. Performance was also abnormal in a precued choice reaction time task in which the precue accurately predicted the nature of the cue and hence the movement that would be required. In both these tasks, the nature of the movement required was highly predicted and performance was impaired. However, in an implicit motor learning task (learning of a visuomotor transformation) and in a precued choice reaction time task, when the precue was only partially predictive or was not predictive of the cue, performance of patients was normal. Importantly, the degree of deficit in patients (e.g., a mean 40 ms slowing of reaction time to valid cues in the precued reaction time task) is much less than that reported in previous studies of malingered poor performance [14]. Instead the implication is that when highly predicted movement is performed there is an opportunity for attention towards movement production. This does not normally occur in healthy people [15] but does in patients with FMD, and as a result, just as when healthy people are explicitly instructed to pay attention to movement [15], movement production is impaired. This work fits with the previous finding of impairment in movement in patients with functional weakness when movement was primed by a consciously perceived cue, compared with normal performance when movement was primed by a cue that was not consciously perceived [16].

Complexity regarding the nature of attention and FMD has been added by recent work that compared positron emission tomography of regional cerebral blood flow in a small cohort of patients with ‘fixed’ functional dystonia and genetically characterized primary dystonia as well as healthy controls [17]. Both patient groups had abnormally increased blood flow during movement in right dorsolateral prefrontal cortex. Functional dystonia patients had reduced blood flow in primary motor cortex and increased blood flow in basal ganglia and cerebellum with an opposite pattern seen in patients with genetic primary dystonia. The authors suggest that although the prefrontal activation could represent abnormal movement-related attention, present in both dystonia groups, the abnormal subcortical activations in functional patients could reflect additional problems with self-directed attention/monitoring, perhaps related to frontosubcortical circuits mediating motor attention, or even reflecting a contribution from connected limbic structures.

## BELIEFS

A recent study has explored an abstract probabilistic reasoning task in patients with FMD [18^▪^]. This version of the ‘jumping to conclusions’ task presents subjects with two jars full of coloured beads, one with 80% blue and 20% red beads and the other with 80% red beads and 20% blue beads. The jars are hidden and subjects are presented with a predetermined sequence of beads and asked when they are certain which jar the beads are being drawn from. Patients with FMD made a judgement after significantly fewer draws than controls, often after just one or two beads had been displayed. The authors hypothesized that such a reasoning style coupled with sensory data occurring during a physical triggering event might produce inappropriate updating of expectations regarding future sensory data, for example an expectation of pain, abnormal movement or weakness, which might drive future physical symptoms. The same style of rapid decision-making on this task has previously been reported in patients with delusional beliefs [19], for example, in the context of schizophrenia. This work makes a tentative but interesting parallel therefore between the neurobiology of delusions [20] and of FMD.

Expectations or prior beliefs play an important role in altering sensory experience. This is, for example, the basis of placebo effects. An important effect of beliefs altering sensory experience has been reported in patients with functional tremor. Patients with functional and organic tremor were asked to wear a wristwatch-like accelerometer, which constantly recorded and stored data on tremor duration over 5 days [21]. During this time, patients also completed diaries rating how much of the time they felt they had tremor. Both patient groups overestimated the amount of time they had tremor, but functional tremor patients did this to a much greater extent than patients with organic tremor. They had on average 30 min of tremor a day, but rated themselves affected by tremor 80–90% of the waking day. This suggests a significant exaggeration of a natural bias in tremor patients to overestimate tremor duration. One hypothesis to explain this is that there is a shift in the interaction between sensory data from the tremoring limb and ‘top-down’ expectations/predictions, so that with a very strong top-down prediction of constant tremor in patients with functional tremor, periods without tremor are simply not perceived.

## AGENCY

When healthy subjects are asked to judge the timing of a self-paced movement or of an auditory tone, there is a difference in judgement depending on whether the movement or tone occurs separately, or if the movement is believed by the subject to cause the tone to happen. In the latter context, the perceived timings of the two events move closer to each other (the movement is perceived to happen later and the tone earlier) [22]. The net effect of this perceptual distortion is to ‘bind’ the two events together. This effect does not occur when the movement is externally generated (for example by a transcranial magnetic stimulus over the motor cortex causing the finger to move), and it is hypothesised that ‘action–effect temporal binding’ indexes sense of agency for movement [22]. In a recent study, this action–effect binding was explored with an additional experimental manipulation in which different pitches of tone were preconditioned with pictures of faces with different emotional valence (happy, neutral, sad) [23^▪^]. There was no effect of this preconditioning on performance, but overall patients with FMD showed reduced action–effect binding compared with healthy controls.

This work fits with a previous functional imaging study in patients with functional tremor in which activations related to habitual tremor were compared with activations occurring when patients deliberated mimicked their tremor [24]. There was a significant hypoactivity found during habitual tremor production in the right temporoparietal junction. This area is hypothesized to be a key node in the network underlying segregation of self-generated vs. externally generated sensation, and hence also highly relevant to the process underlying sense of agency. On the same theme, a previous study employing a widely used (but also often criticised) paradigm thought to assess aspects of sense of agency – the Libet paradigm – found patients with FMD to lack the normal period of subjective sense of intention to move prior to a self-paced movement compared with healthy controls [25].

## FROM CONCEPTS TO NEUROBIOLOGICAL MODELS

The discussion above suggests three key concepts that need to be integrated into any model that seeks to explain the generation of FMD. In addition, one would hope that any model could incorporate a role for known precipitating factors and risk factors for development of FMD, as well as being broad enough to account for the known common co-occurrence of different functional neurological symptoms in the same patient.

Voon *et al.*[26], against the background of a number of functional imaging and behavioural studies in FMD [24,27^▪▪^,^28^], have proposed a model for how FMD might be generated. The key concept here is of a ‘previously mapped conversion motor representation’, that is, a (conditioned) pattern of movement established perhaps by a previous triggering event. The commonly reported presence of physical precipitating factors at onset such as injury could provide a relevant trigger for development of a ‘conversion motor representation’. The additional emotional arousal that is often reported in the background and/or at the time of onset could certainly increase the salience of sensory information arising during a physical trigger and facilitate this process. Functional imaging studies from this group have provided evidence for hypoactivity in areas usually associated with action selection [e.g., supplementary motor area (SMA)], as well as abnormally strong connectivity between limbic structures (e.g., amygdala) and SMA [26,28]. The proposal is that in an arousing context, the previously mapped conversion motor representation is activated in part because of the abnormal functional connectivity between limbic structures and SMA, and cannot be inhibited because there is a disconnection between SMA and areas (prefrontal cortex for example) that could usually inhibit unwanted action. The result is a movement that arises without a normal prediction of its sensory consequences (efference copy) and is therefore (mis)interpreted by patients as without agency and therefore not self-generated [26].

Edwards *et al.*[29^▪▪^] have proposed a model that employs Bayesian theories of brain function (active inference) to explain functional sensory and motor symptoms, including FMD. This model relies on the instigation of an abnormal ‘prior’ or expectation, proposed to reside in an intermediate level of the cortical hierarchy such as SMA (for those with motor symptoms). It is suggested that a variety of factors could be relevant to the formation of this abnormal prior, likely to be different for different patients, including physical precipitants that provide novel sensory data about the self, panic responses at the time of symptom onset, affective disorders, personal and cultural illness beliefs, and decision-making styles such as those found in the ‘jumping to conclusions’ task. The proposal is that self-directed attention would, via increasing the precision (weight) applied to the prior over relevant ‘bottom-up’ sensory data, cause movement or percepts in keeping with the prior. Importantly, as higher cortical regions (for example prefrontal regions) mediating self-directed attention carry no prediction about the content of the prior, the resulting movement or percept is experienced as an unwilled phenomenon, which is then quite rationally attributed by the patient as a symptom.

## CONCLUSION

There are clear areas of overlap between these models, and indeed with previous models, for example, Brown's cognitive model [30] of medically unexplained symptoms in which he proposes a role for ‘rogue representations’, and indeed going back as far as Russell Reynolds’ proposal in the mid 19th century that beliefs about symptoms can involuntarily alter movement or perception [31]. The advancement of recent models is to place FMD within a testable, biologically plausible framework. This framework still includes an important potential role for the ‘presumed psychological factors’ discussed at the beginning of this review, but also allows considerable flexibility in the manner in which symptoms might be triggered in individual patients. This shift in emphasis has relevance for diagnostic explanations for patients [32] and treatment development, and it is hoped will eventually improve the provision of appropriate care and long-term prognosis for patients with FMD and other functional neurological symptoms.

## Acknowledgements

None.

### Conflicts of interest

There are no conflicts of interest.

## REFERENCES AND RECOMMENDED READING

Papers of particular interest, published within the annual period of review, have been highlighted as:

▪ of special interest
▪▪ of outstanding interest

Additional references related to this topic can also be found in the Current World Literature section in this issue (p. 455).

## References

[1] Ellenstein A Kranick SM Hallett M An update on psychogenic movement disorders. Curr Neurol Neurosci Rep 2011; 11:396–403 21559795 10.1007/s11910-011-0205-z PMC4747629

[2] Kranick S Ekanayake V Martinez V Psychopathology and psychogenic movement disorders. Mov Disord 2011; 26:1844–1850 21714007 10.1002/mds.23830 PMC4049464

[3] Stone J Edwards MJ How ‘psychogenic’ are psychogenic movement disorders? Mov Disord 2011; 26:1787–1788 21761457 10.1002/mds.23882

[4] Espay AJ Goldenhar LM Voon V Opinions and clinical practices related to diagnosing and managing patients with psychogenic movement disorders: an international survey of Movement Disorder Society members. Mov Disord 2009; 24:1366–1374 19425106 10.1002/mds.22618

[5] Stone J LaFrance WC Jr Brown R Conversion disorder: current problems and potential solutions for DSM-5. J Psychosom Res 2011; 71:369–376 22118377 10.1016/j.jpsychores.2011.07.005

[6] Edwards MJ Bhatia KP Functional (psychogenic) movement disorders: merging mind and brain. Lancet Neurol 2012; 11:250–260 22341033 10.1016/S1474-4422(11)70310-6

[7] Gupta A Lang AE Psychogenic movement disorders. Curr Opin Neurol 2009; 22:430–436 19542886 10.1097/WCO.0b013e32832dc169

[8] Morgante F Edwards MJ Espay AJ DISMOV-SIN Study Group On Psychogenic Movement Disorders Diagnostic agreement in patients with psychogenic movement disorders. Mov Disord 2012; 27:548–552 22488862 10.1002/mds.24903 PMC3675653

[9] Schwingenschuh P Katschnig P Seiler S Moving toward ‘laboratory-supported’ criteria for psychogenic tremor. Mov Disord 2011; 26:2509–2515 21956485 10.1002/mds.23922 PMC3664413

[10] van Poppelen D Saifee TA Schwingenschuh P Attention to self in psychogenic tremor. Mov Disord 2011; 26:2575–2576 22025317 10.1002/mds.23911

[11] Friston K The free-energy principle: a rough guide to the brain? Trends Cogn Sci 2009; 13:293–301 19559644 10.1016/j.tics.2009.04.005

[12] Haggard P Chambon V Sense of agency. Curr Biol 2012; 22:R390–R392 22625851 10.1016/j.cub.2012.02.040

[13▪] Pareés I Kassavetis P Saifee TA Failure of explicit movement control in patients with functional motor symptoms. Mov Disord 2013; 28:517–523 23408383 10.1002/mds.25287

[14] Willison J Tombaugh TN Detecting simulation of attention deficits using reaction time tests. Arch Clin Neuropsychol 2006; 21:41–52 16280230 10.1016/j.acn.2005.07.005

[15] Jueptner M Stephan KM Frith CD Anatomy of motor learning. I. Frontal cortex and attention to action. J Neurophysiol 1997; 77:1313–1324 9084599 10.1152/jn.1997.77.3.1313

[16] Roelofs K van Galen GP Eling P Endogenous and exogenous attention in patients with conversion paresis. Cogn Neuropsychol 2003; 20:733–745 20957591 10.1080/02643290342000069

[17] Schrag AE Mehta AR Bhatia KP The functional neuroimaging correlates of psychogenic versus organic dystonia. Brain 2013; 136 (Pt 3):770–781 23436503 10.1093/brain/awt008 PMC3580272

[18▪] Pareés I Kassavetis P Saifee TA ’Jumping to conclusions’ bias in functional movement disorders. J Neurol Neurosurg Psychiatry 2012; 83:460–463 22338028 10.1136/jnnp-2011-300982

[19] Garety PA Hemsley DR Wessely S Reasoning in deluded schizophrenic and paranoid patients. Biases in performance on a probabilistic inference task. J Nerv Ment Dis 1991; 179:194–201 2007889 10.1097/00005053-199104000-00003

[20] Corlett PR Taylor JR Wang XJ Toward a neurobiology of delusions. Prog Neurobiol 2010; 92:345–369 20558235 10.1016/j.pneurobio.2010.06.007 PMC3676875

[21] Pareés I Saifee TA Kassavetis P Believing is perceiving: mismatch between self-report and actigraphy in psychogenic tremor. Brain 2012; 135:117–123 22075068 10.1093/brain/awr292

[22] Haggard P Clark S Kalogeras J Voluntary action and conscious awareness. Nat Neurosci 2002; 5:382–385 11896397 10.1038/nn827

[23▪] Kranick SM Moore JW Yusuf N Action-effect binding is decreased in motor conversion disorder: Implications for sense of agency. Mov Disord 2013; [Epub ahead of print] 10.1002/mds.25408 PMC3701023 23494975

[24] Voon V Gallea C Hattori N The involuntary nature of conversion disorder. Neurology 2010; 74:223–228 20083798 10.1212/WNL.0b013e3181ca00e9 PMC2809033

[25] Edwards MJ Moretto G Schwingenschuh P Abnormal sense of intention preceding voluntary movement in patients with psychogenic tremor. Neuropsychologia 2011; 49:2791–2793 21683724 10.1016/j.neuropsychologia.2011.05.021

[26] Voon V Brezing C Gallea C Hallett M Aberrant supplementary motor complex and limbic activity during motor preparation in motor conversion disorder. Mov Disord 2011; 26:2396–2403 21935985 10.1002/mds.23890 PMC4162742

[27▪▪] Voon V Ekanayake V Wiggs E Response inhibition in motor conversion disorder. Mov Disord 2013; 28:612–618 23554084 10.1002/mds.25435 PMC4096145

[28] Voon V Brezing C Gallea C Emotional stimuli and motor conversion disorder. Brain 2010; 133 (Pt 5):1526–1536 20371508 10.1093/brain/awq054 PMC2859149

[29▪▪] Edwards MJ Adams RA Brown H A Bayesian account of ’hysteria’. Brain 2012; 135:3495–3512 22641838 10.1093/brain/aws129 PMC3501967

[30] Brown RJ Psychological mechanisms of medically unexplained symptoms: an integrative conceptual model. Psychol Bull 2004; 130:793–812 15367081 10.1037/0033-2909.130.5.793

[31] Reynolds R Paralysis and other disorders of motion and sensation dependent on idea. BMJ 1869; i:483–485 10.1136/bmj.2.462.483 PMC2261202 20745663

[32] Stone J Edwards M Trick or treat? Showing patients with functional (psychogenic) motor symptoms their physical signs. Neurology 2012; 79:282–284 22764261 10.1212/WNL.0b013e31825fdf63

